# Distribution and Release Characteristics of Phosphorus in a Reservoir in Southwest China

**DOI:** 10.3390/ijerph16030303

**Published:** 2019-01-23

**Authors:** Yuanming Wang, Kefeng Li, Ruifeng Liang, Shiqing Han, Yong Li

**Affiliations:** 1 State Key Laboratory of Hydraulics and Mountain River Engineering, Sichuan University, Chengdu 610065, China; wangyuanming1991@126.com (Y.W.); kefengli@scu.edu.cn (K.L.); ruifengliangscu@126.com (R.L.); 2 China University of Petroleum-Beijing at Karamay, Karamay 834000, China; HsqExcellent@163.com

**Keywords:** phosphorus, distribution, release characteristics, reservoir, sediment, overlying water

## Abstract

Dam construction changes the nutrient transport of a river system. Phosphorus is an important fundamental material in the global biochemical cycle and is always a limiting factor in the primary productivity of reservoirs. Extending the study of phosphorus in reservoirs is necessary given the dam construction in southwest China. Zipingpu Reservoir was chosen as the research site in this study. The form and distribution of phosphorus in the reservoir’s surface sediments and overlying water were analyzed. The results showed that overall, the total phosphorus (TP) content of surface sediments in the Zipingpu Reservoir decreased from the tail to the front of the dam. The TP content ranged from 682.39 to 1609.06 mg/kg, with an average value of 1121.08 mg/kg. The TP content at some sampling points was affected by exogenous input. Inorganic phosphorus (IP) was the main form of phosphorus in surface sediments and had a proportion of 89.38%. Among the forms of IP, the content of Ca-P was larger than that of O-P; Ex-P, Fe-P, and Al-P had the lowest contents. Particulate phosphorus (PP) was the main form of phosphorus in the overlying water of the Zipingpu Reservoir and was strongly affected by hydrodynamic conditions. The content of total dissolved phosphorus (TDP) in the overlying water was relatively low. To further understand the risk of phosphorus release in the surface sediments in the reservoir, the rate and flux of phosphorus exchange at the sediment-overlying water interface were investigated through laboratory experiments. The results showed that both water temperature and pH significantly affected the sediment release rate, but the influence of water temperature was more significant. Acidic and alkaline conditions were conducive to the release of phosphorus from sediment, while a neutral environment was not. The release rate significantly increased with increasing water temperature, and a positive linear relationship was found between these two parameters. The sediment exhibited absorption characteristics when the water temperature was extremely low and exhibited releasing characteristics at a high temperature. These results could provide a theoretical basis for the management and protection of reservoir water environments.

## 1. Introduction

Dam construction changes the hydrologic and hydraulic elements of the river system, and nutrient transport is also altered. A large amount of nutrients is stored in reservoirs through biological adsorption, physical settlement, and other functions; this storage results in a decrease in nutrient output downstream of the dam [1,2]. The retention of nutrients in reservoirs can impact the total amount delivered from land to sea and change the water quality of reservoirs. Phosphorus is an important fundamental material in the global biochemical cycle and is always a limiting factor of primary productivity in reservoirs [3,4].

Phosphorus in sediment can be classified as organic or inorganic. The components of organic phosphorus (OP) are difficult to identify and isolate; thus, many studies have treated OP as a single form of phosphorus. Generally, inorganic phosphorus (IP) is present in the form of phosphorus bound to iron (Fe-P), aluminum (Al-P) and calcium (Ca-P), as well as in the form of exchangeable phosphorus (Ex-P), occluded P (O-P), and others [5,6]. Ex-P mainly refers to phosphorus that is adsorbed to or coprecipitated with particles in the sediment, such as active clay minerals, iron oxides, manganese oxides, hydroxides, and others [7]. Among the different forms of phosphorus, Ex-P easily enters water and is then used or adsorbed by organisms. The content of Ex-P is usually not high, but this form of phosphorus is most easily used by organisms. Fe-P and Al-P can be used by aquatic organisms when present in water in certain circumstances. Fe-P is susceptible to environmental changes. Ferric ion always exists under aerobic conditions and easily combines with phosphorus to precipitate phosphate. Under anaerobic conditions, insoluble ferric hydroxide can easily convert into soluble ferric hydroxide, which increases the risk of water eutrophication. Many compounds in water can be used as extractants to dissolve Al-P from sediments [5]. Ca-P in sediments mainly exists in the form of calcium phosphate, which is poorly soluble and cannot readily be used by living creatures. However, a sufficient carbon dioxide content in the water can increase the solubility of Ca-P and promote its release. O-P, which includes some Al-P and Ca-P, is a phosphorus salt and is mainly covered by a layer of ferric oxide film. O-P is considered to be biologically unavailable and can persist for a long time [8].

Sediment plays an important role in the biogeochemical circulation of phosphorus. Sediment can accept phosphorus from overlying water and release phosphorus to overlying water. Research in Clear Lake showed that over 3500 t of phosphorus was stored within a 10 mm layer of surface sediment. Every year, the exogenous input of phosphorus to Clear Lake is approximately 160 t, and 550–700 t of P is involved in the inner circulation of the lake [9]. In some lakes and reservoirs in northern China, the exogenous pollution sources are well controlled. Studies have shown that phosphorus is continuously released into overlying water when sediments at the bottom of a reservoir have a sufficient phosphorus content. In this situation, endogenous phosphorus becomes an important cause of water quality deterioration [10].

Many scholars have studied the distribution and release characteristics of phosphorus in rivers, lakes and marine [7,11,12,13]. Some research has even been conducted in reservoirs in China, most of them concentrated in the downstream of Yangtze River, or other river basins [14,15]. Few studies on the distribution and release characteristics of phosphorus have been conducted in a reservoir in southwest China. In recent years, many hydropower projects have been planned or built in southwest China [16,17,18]. These reservoirs always have great water depth (i.e., over 200 m or even as deep as 300 m) and small surface width, which make them have different distribution and release characteristics of phosphorus than other waters. To learn the distribution and release characteristics of phosphorus in reservoirs in southwest China, the Zipingpu Reservoir was chosen as the research site in this study. The form and distribution of phosphorus in the surface sediments of the reservoir were analyzed in this study, and the phosphorus content in the overlying water was measured. To further understand the risk of phosphorus release from the surface sediments in the reservoir, the rate and flux of phosphorus exchange at the sediment-overlying water interface were investigated through laboratory experiments. These results can provide a theoretical basis for the management and protection of reservoir water environments.

## 2. Materials and Methods

### 2.1. Study Site

The Zipingpu Reservoir is in the upper Min River northwest of Chengdu City. The reservoir is a water conservancy project with comprehensive benefits, such as water supply and irrigation, as well as power generation, flood control and environmental protection. The Zipingpu Reservoir, which has a total capacity of 11.12 × 10^8^ m^3^ and a regulating capacity of 7.74 × 10^8^ m^3^, is the managed water source serving the famous Dujiangyan irrigation district. The normal water level is 877 m, and the minimum operating level is 817 m. The Shouxi River, a tributary of the dam, joins 12 km upstream.

### 2.2. Sample Collection

Samples of overlying water were collected from March 2016 to February 2017 to analyze the form and distribution of phosphorus in the Zipingpu Reservoir. A self-made sampling system was used to collect the overlying water at the bottom of the reservoir [19]. The temperature, pH, and dissolved oxygen (DO) were also measured at the same time. The overlying water samples were placed in clean polyethylene bottles and stored frozen in the laboratory.

**Figure 1:**
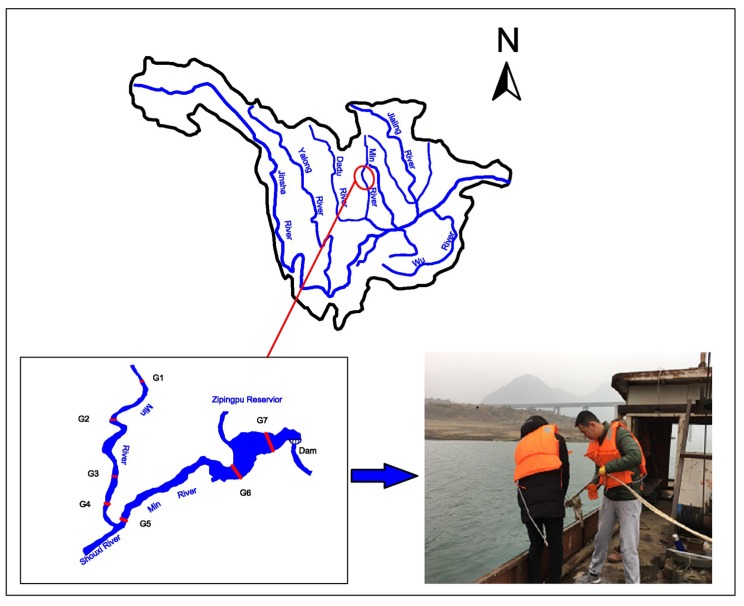
Location of the Zipingpu Reservoir and field sampling site.

Surface sediments of the reservoir were collected in April 2016 to analyze the form and distribution of phosphorus and in February 2017 to conduct laboratory experiments on the rate and flux of phosphorus exchange. A device for sediment sampling was used to sample the surface sediments. The first 20 cm sediments of the bottom were collected and then taken to the laboratory and stored frozen. The sampling points are shown in Figure 1 and Table 1.

### 2.3. Analysis of Overlying Water and Surface Sediments

First, the unfiltered overlying water was digested, and then TP was measured by ammonium molybdate spectrophotometry. For the measurement of TP in surface sediments, after initial drying, the sediments were first spread on a clean plastic film and milled through a 100-mesh sieve. Stones and debris from animals and plants were removed during milling. The surface sediments were digested by concentrated sulfuric acid and perchlorate, and the TP content was determined in a molybdate-antimony-scandium color agent. Different forms of P, including Ex-P, Al-P, Ca-P, Fe-P, O-P and OP, were quantified in the sediments through a sequential extraction procedure which was described by [20].

### 2.4. Laboratory Experiments of the Rate and Flux of P

From the reservoir tail to the dam site, the reservoir can be divided into a river section, transition section, and reservoir section. Sampling points G2, G4 and G6 represented the river section, transition section, and reservoir section, respectively, in the laboratory experiments. Laboratory experiments investigating the rate and flux of P considered the temperature and pH: five temperature levels (5, 9, 12, 15 and 19 °C) and three pH levels (6 ± 0.2, 7 ± 0.2 and 8 ± 0.2) were used. Considering the sampling amount of surface sediments and overlying water, and referring to previous studies, we set the water-sediment thickness ratio as 6:1 [21,22]. The experiments were conducted in an adjustable-temperature thermostatic chamber.

For each case, sediments collected from the Zipingpu Reservoir were placed in a cubic container made of glass (25 cm × 25 cm × 40 cm), and then overlying water that had been filtered through a 0.45 μm membrane was slowly added into the cubic container by siphoning. The pH was adjusted by Na_2_CO_3_ and HCl. On each day, 25 mL of water was collected by a pipette from 2 cm above the sediments and then used for TP measurements. The same volume of filtered overlying water was immediately added to the cubic container to replace the removed sample.

### 2.5. Statistical Analysis

The release rate of TP was used to describe the release characteristics in the laboratory experiment and is expressed as follows:

(1) $$K_{i}=[(C_{j}-C_{0})V-\sum_{j=1}^{n}(C_{j-1}-C^{\prime })V^{\prime }]/(A^{\prime }\cdot t)$$

where *Κ_i_* is the release rate of TP in case *i*, mg/(m^2^·d); *C_j_* and *C_j_*_− 1_ are the TP concentrations measured in sample *j* and *j* − 1, respectively, mg/L; *C*_0_ is the TP concentration of the overlying water at the beginning of the experiment, mg/L; *C*’ is the TP concentration of the filtered overlying water, mg/L; *V* is the volume of the overlying water, L; *V*’ is the volume of the filtered overlying water, L; *À* is the area of the interface between the sediment and overlying water, m^2^; and *t* is the duration of the experiment, d.

One-way analysis of variance (ANOVA) was used to test differences in means among TP values and was followed by a post hoc multiple comparison test (least-significant difference) to determine where those differences occurred. Two-way ANOVA was performed to determine the effects of two factors and their interaction. Post hoc multiple comparisons were performed using the S-N-K test. The level of significance was set at *p* < 0.05.

## 3. Results

### 3.1. Spatial-Temporal Distribution of Phosphorus in Sediments

**Figure 2:**
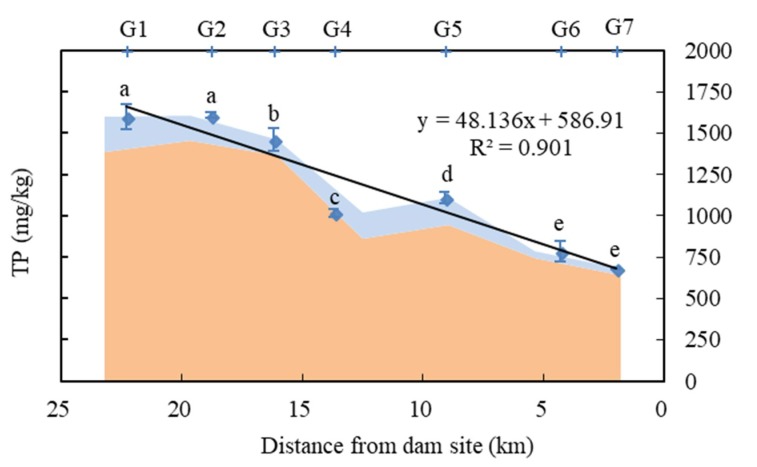
The concentration of phosphorus at each sampling site. The squares represent total phosphorous (TP) values, which are shown as means ± SD (*n* = 4). The letters above the squares indicate the results from a post hoc multiple comparison test (least-significant difference test); mean values that do not share a common lowercase letter are significantly different (*p* < 0.05). The area in orange represents IP, and that in light blue represents OP.

As shown in Figure 2, the TP content in surface sediments at each sampling point ranged from 682.39-1609.06 mg/kg, and the average value was 1121.08 mg/kg. The TP content significantly decreased from the tail to the front of the Zipingpu Dam, except for points G2 and G5 (one-way ANOVA: *F* = 199.82, *df*1 = 6, *df*2 = 19, *p* < 0.001). A linear relationship was found between the TP content and the distance from the dam site (R^2^ = 0.901). Inorganic phosphorous accounted for a major portion (86.5–94.5%) of the TP at each site, and the IP content ranged from 642.12–1456.87 mg/kg. The average proportion of OP relative to TP was 10.6%.

The contents of different forms of IP are shown in Table 2. Ca-P accounted for a major portion (61.2–88.4%) of the TP at each site, and the content of Ca-P ranged from 489.09–1264.72 mg/kg. The content of O-P as a proportion of TP ranked second at each site and ranged from 9.6–30.8%. Ex-P, Al-P, and Fe-P accounted for a minority of TP at each site. Two-way ANOVA showed that both the form and the sampling site had a significant impact on the phosphorus content (Table 3). The contents at sites G4-G7 significantly differed from those at G1-G3. The contents of Ex-P, Al-P, and Fe-P were significantly smaller than the other forms of phosphorus; O-P had the next highest content, and Ca-P had the highest content.

### 3.2. Spatial-Temporal Distribution of Phosphorus in Overlying Water

**Figure 3:**
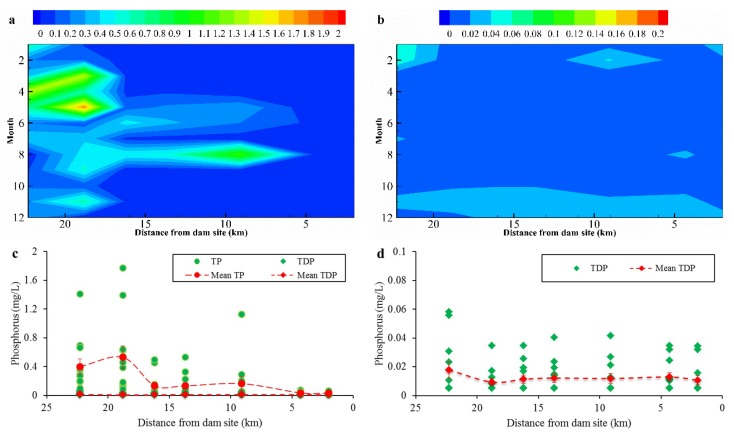

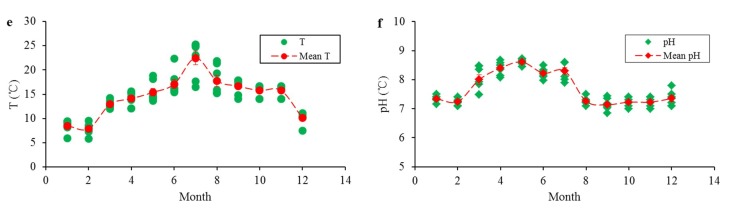
Environmental parameters of the overlying water in the Zipingpu Reservoir. (**a**) and (**b**) represent the spatial-temporal distribution of TP and TDP, respectively. Circles in (**c**) and (**d**) indicate TP and TDP values at different sampling sites and in different months. Circles in (**e**) and (**f**) indicate the temperature and pH of overlying water at different mouths. Both mean values with SE and individual values are exhibited in (**c**–**f**).

For the overlying water at the bottom of the reservoir, its TP was mainly distributed in the river section, which was more than 15 km away from the dam site, and the concentration ranged from 0.015–1.770 mg/L (Figure 3a). The total dissolved phosphorus (TDP) was also determined in this study: the TDP contents at most sampling points were lower than the detection limit (0.005 mg/L) in March, May, and June. Only one sample had a detectable content in April, August, and September (Figure 3b). TDP accounted for only a small part of TP (6.1% of TP), which indicated that particulate phosphorus (PP) was the major form of phosphorus in the overlying water in the Zipingpu Reservoir (Figure 3c,d). The water temperature of the reservoir ranged from 5.8 (February) to 25.3 °C (July), and the pH ranged from 6.84 to 8.73 (Figure 3e,f). The reservoir exhibited alkaline characteristics from March to July and had a neutral environment in other months.

### 3.3. Characteristics of Phosphorus Exchange at the Sediment-Water Interface

**Figure 4:**
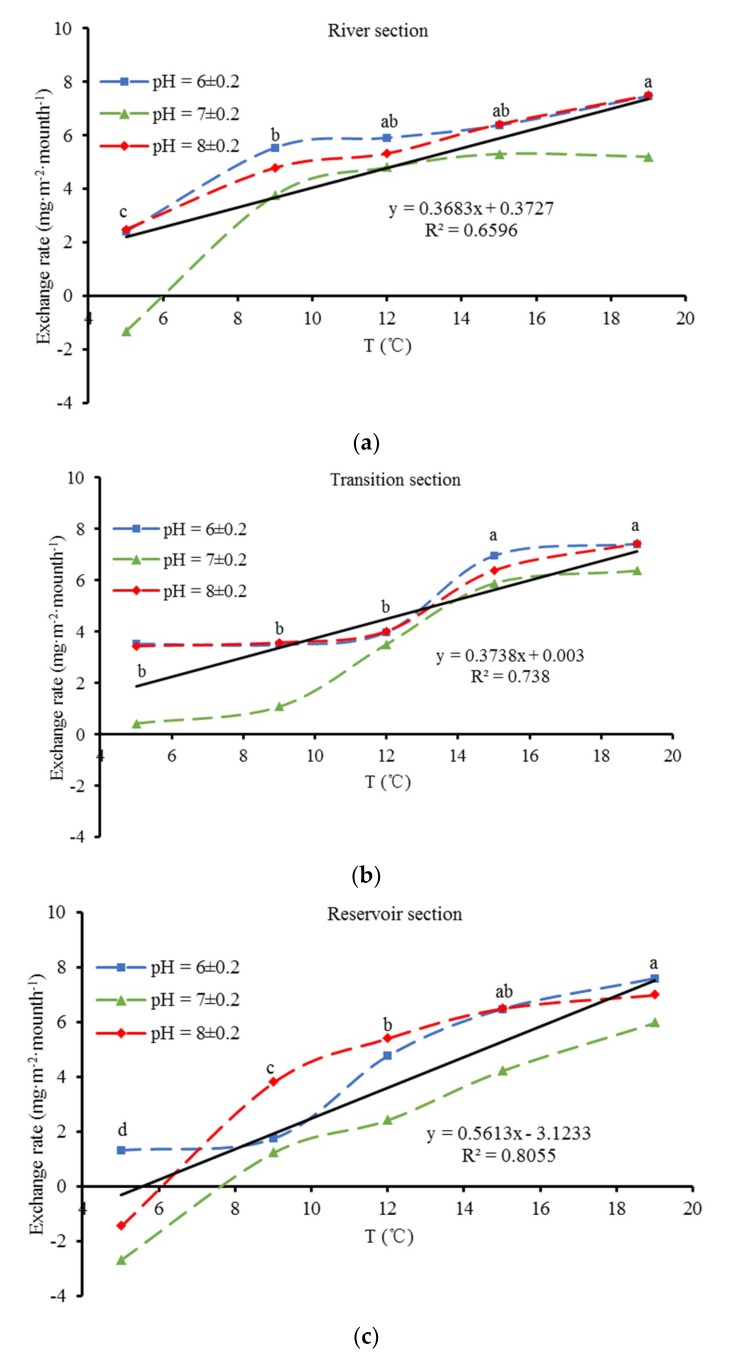
Exchange rate of phosphorus with varying temperature and pH values at different sections. Letters above the values indicate the results from a post hoc multiple comparison test (S-N-K test); values that do not share a common lowercase letter indicate a significant difference between temperatures (*p* < 0.05).

Two-way ANOVA showed that the release rate of sediment was significantly affected by pH (Table 4), and the release rate was lower at a pH of 7 ± 0.2 than those at pH 6 ± 0.2 and 8 ± 0.2 (Figure 4), which indicated that acidic and alkaline conditions were conducive to the release of phosphorus from sediment, while a neutral environment was not. The impact of pH on phosphorus release varies with the content and form of phosphorus in the sediment [23,24]. Phosphorus in Fe-P and Al-P is stable under acidic conditions, while phosphorus in Ca-P is easily released when the H^+^ concentration is high enough. Moreover, microorganisms produce CO_2_ in the process of organic matter degradation, which is also conducive to the release of phosphorus from Ca-P. When the pH of the overlying water reaches approximately 7, Al^3+^ is hydrolyzed to colloidal Al(OH)_3_. Al(OH)_3_ has a stronger absorption capacity than HPO_4_^2−^ and H_2_PO_4_^−^. The TP content decreases with absorption by colloidal Al(OH)_3_ at a pH of 7. Under alkaline conditions, phosphorus release increases with the conversion of OH^−^ in Al-P and Fe-P to H_2_PO_4_^−^. In contrast, the surface of variable-charge colloids in sediments always exhibits a negative charge under alkaline conditions; thus, the absorption capacity of sediments for HPO_4_^2−^ in the overlying water decreases, and then an increasing TP content in the overlying water follows [25,26].

The release rate was also significantly affected by water temperature (Table 4) and significantly increased with increasing water temperature (Figure 4). A positive linear relationship was found between the release rate and water temperature in each section (Figure 4). Two-way ANOVA showed that water temperature had a more significant influence on the release rate than pH (Table 4). An appropriate temperature increases the microbial activity and decomposition rate of organic matter in sediments, which promotes the release of OP into overlying water. An increase in temperature weakens the mineral adsorption capacity for phosphorus, thus enabling the phosphorus in the sediments to enter the overlying water more easily [27]. Increasing temperature can also promote biological disturbance, anaerobic transformation, and mineralization, which results in a reductive state at the sediment surface and the transformation of Fe^3+^ to Fe^2+^. In this situation, phosphorus is released from iron orthophosphate and iron hydroxide, thus accelerating the release of phosphorus from sediments [28].

Under a neutral environment with a water temperature of 5 °C in the river and reservoir sections, the TP release rates of sediment were −1.33 and −2.71 mg∙m^−2^∙month^−1^, respectively; these release rates indicated absorption characteristics and resulted in decreases in the TP concentration of the overlying water. The sediment in the reservoir section also exhibited absorption characteristics under an alkaline environment with a water temperature of 5 °C. The sediment in the transition section exhibited release characteristics at different temperatures. All surface sediments were in a release state at a temperature of 9–19 °C.

## 4. Discussion

### 4.1. Phosphorus in Sediments and Overlying Water

**Figure 5:**
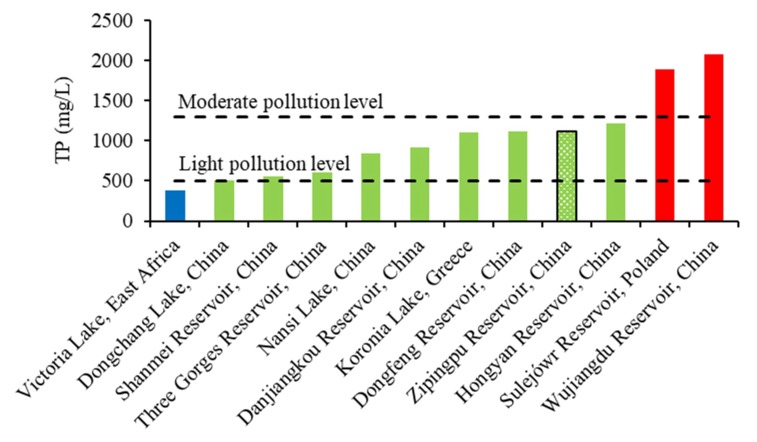
Sediment TP contents in different reservoirs and lakes.

Sediment is an important medium for phosphorus removal and regeneration. The endogenous phosphorus load in surface sediment weakens the water governance effect and may become significant when the exogenous phosphorus is effectively controlled. The TP of sediment has been investigated in many reservoirs and lakes (Figure 5). Compared with these previous investigations, Zipingpu Reservoir has a relatively high level of TP in surface sediment [14,29,30,31,32,33,34,35,36]. Sediment pollution can be divided into three levels according to TP content: light pollution (TP < 500 mg/kg), moderate pollution (500 mg/kg < TP < 1300 mg/kg), and heavy pollution (TP > 1300 mg/kg) [37,38]. The TP content of the surface sediment in the Zipingpu Reservoir ranged from 682.39–1609.06 mg/kg and had an average value of 1121.08 mg/kg. Sediment pollution at the tail of the Zipingpu Reservoir reached a high level, and at the middle and front of the dam, sediment pollution reached a moderate level. 

The TP content significantly decreased from the tail to the front of Zipingpu Dam, except at sites G2 and G5. The TP content at G2 was slightly higher than that at G1. This difference is mainly caused by wastewater from Yingxiu, a county downstream of G2 that was reconstructed after being seriously damaged by the Wenchuan earthquake. Due to the Shouxi tributary, the TP content at G5 was significantly higher than that at G4.

**Figure 6:**
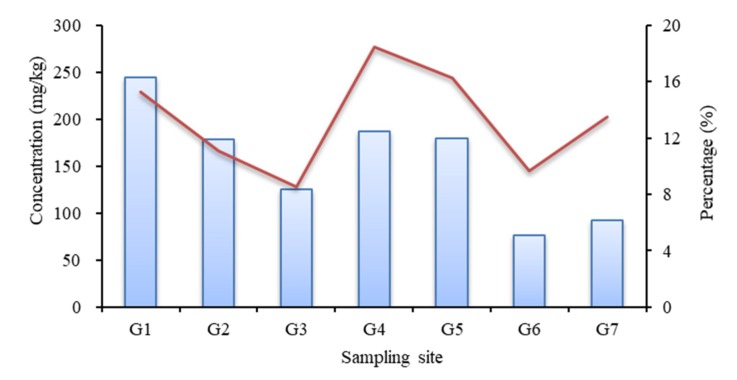
The concentration of potential bioavailable phosphorus (blue bars) and its proportion (red line) relative to TP in the Zipingpu Reservoir.

The TP content can only reflect the accumulation of phosphorus in sediments and cannot directly show the release risk or biological stability. It is important to further analyze various forms of phosphorus in the sediments in the Zipingpu Reservoir. Different forms of phosphorus in sediments have different biochemical behaviors and bioavailabilities, and the potential influence of sediment on the water quality of overlying water cannot be ignored. Some phosphorus can be released from sediments and can be used by aquatic organisms; this fraction is defined as potential bioavailable phosphorus [39]. The water pH in the Zipingpu Reservoir was weakly alkaline, and Ex-P, Al-P, Fe-P, and OP in the Zipingpu Reservoir can be regarded as potential bioavailable phosphorus. As shown in Figure 6, the proportion of potential bioavailable phosphorus relative to TP in the Zipingpu Reservoir ranged from 8.53 (G3) to 18.45% (G4). The tail and middle of the reservoir had a relatively high content of potential bioavailable phosphorus, with values ranging from 76.15–244.20 mg/kg. The potential bioavailable phosphorus in the Zipingpu Reservoir was present at a light pollution level. Under conditions typical of a weakly alkaline water body, the potential release risk of phosphorus in the surface sediments of the Zipingpu Reservoir was small.

PP was the major form of phosphorus in overlying water in the Zipingpu Reservoir. Dynamic water conditions associated with reservoir operation were the main factors controlling phosphorus transport and subsidence. We speculate that some solid-phase phosphorus settled out with sediment particles before reaching the middle or front of the dam after entering the reservoir, which caused high concentrations of TP and PP at the tail of the reservoir.

### 4.2. Exchange Characteristics of Phosphorus at the Sediment-Water Interface

Reservoir sediment is an important material for the accumulation of nutrients. In addition to external sources (such as the surrounding environment and reservoir inflow), some sources are based on sediment exchange. Nutrients are released into the overlying water by convection and diffusion processes when the reservoir environment changes, which causes reservoir sediment to become a source of endogenous pollution.

Temperature had a significant influence on the exchange rate of phosphorus in the surface sediments of the Zipingpu Reservoir. At 5 °C, the sediments in the river section, which had a neutral environment, and the reservoir section, which had an alkaline or neutral environment, existed in an absorption state, while sediments at 5 °C in other conditions existed in a release state. In all cases, all sediments showed a release state at a temperature of 9–19 °C, and the release rate increased with increasing temperature.

## 5. Conclusions

Through the investigation of phosphorus in Zipingpu Reservoir and experiments investigating phosphorus exchange at the sediment-overlying water interface, the following distribution and release characteristics of phosphorus in reservoirs in southwest China can be drawn:

(1) The TP content of surface sediments in reservoirs decreased from the tail to the front of the dam, overall. The IP was the main form of phosphorus in surface sediments.
(2) PP was the main form of phosphorus in the overlying water of the reservoirs and was strongly affected by hydrodynamic conditions. The TDP content in the overlying water was relatively low.
(3) Both water temperature and pH significantly affected the release rate of the sediment, but the influence of water temperature was more significant. Acidic and alkaline conditions were conducive to the release of phosphorus from sediments, while a neutral environment was not. The release rate significantly increased with increasing water temperature. Sediment exhibited absorption characteristics when the water temperature was extremely low and releasing characteristics at a high temperature.

## Figures and Tables

**Table 1:** Detailed information on the sampling points in the Zipingpu Reservoir.

Sampling Site	Distance from Dam Site (km)	Geographic Coordinates	Notes
G1	22.3	N 31′04′03.33″ E 103’29’21.63″	Control section at the reservoir tail
G2	18.8	N 31′02′44.44″ E 103′28′25.93″	Downstream of Yingxiu county
G3	16.2	N 31°00′38.38″ E 103°28′25.16″	The middle of the dam and upstream of the Shouxi River, a tributary of the Zipingpu Reservoir
G4	13.7	N 31°00′08.32″ E 103°28′21.56″	The middle of the dam and upstream of the Shouxi tributary
G5	9.1	N 31°00′25.89″ E 103°29″35.78″	The middle of the dam and downstream of the Shouxi River
G6	4.3	N 31°01′18.66″ E 103°32′47.16′	The middle of the dam and downstream the Shouxi River
G7	2.0	N 31°01′58.90′ E 103°33′37.53′	The front of the dam

**Table 2:** The contents of different forms of inorganic phosphorous (IP) at each sampling site.

Sampling Site	G1	G2	G3	G4	G5	G6	G7
Ex-P (mg/kg)	14.50	15.82	9.09	16.36	11.66	22.61	7.52
Al-P (mg/kg)	4.26	2.81	5.35	2.04	2.16	2.98	4.63
Fe-P (mg/kg)	8.68	7.66	9.22	12.09	2.34	7.42	39.51
Ca-P (mg/kg)	1223.16	1264.72	1171.02	638.72	740.04	489.09	392.80
O-P (mg/kg)	132.68	165.86	167.14	190.23	188.22	222.92	197.65

**Table 3:** Statistical results of two-way ANOVA on the content of each type of IP.

Sources of Variation	df	F	Sig.	Partial Squared
Sampling site	6	10.29	<0.001	0.638
Phosphorus form	4	416.78	<0.001	0.979
Sampling site * Phosphorus form	24	12.85	<0.001	0.898

**Table 4:** Statistical results of two-way ANOVA on the exchange rate of phosphorus at the sediment-water interface at different sections.

Sources of Variation		df	F	Sig.	Partial Squared
River section	pH	2	11.45	0.004	0.741
	T	4	26.97	<0.001	0.931
Transition section	pH	2	9.43	0.008	0.702
	T	4	30.92	<0.001	0.939
Reservoir section	pH	2	7.94	0.013	0.665
	T	4	30.04	<0.001	0.939
